# Structural Racism Operationalized via Adverse Social Events in a Single-Center Neonatal Intensive Care Unit

**DOI:** 10.1016/j.jpeds.2023.113499

**Published:** 2023-05-19

**Authors:** Kayla L. Karvonen, Erica Anunwah, Brittany D. Chambers Butcher, Lydia Kwarteng, Tameyah Mathis-Perry, Monica R. McLemore, Sally Oh, Matthew S. Pantell, Olga Smith, Elizabeth Rogers

**Affiliations:** 1 Department of Pediatrics, University of California San Francisco, San Francisco, CA; 2 California Preterm Birth Initiative, San Francisco, CA; 3 University of California Davis, Sacramento, CA; 4 University of California San Francisco School of Medicine, San Francisco, CA; 5 Morehouse School of Medicine, Atlanta, GA; 6 University of Washington, Seattle, WA; 7 Department of Obstetrics, Gynecology & Reproductive Sciences, University of California San Francisco, San Francisco, CA

## Abstract

**Objective:**

To evaluate structural racism in the neonatal intensive care unit (NICU) by determining if differences in adverse social events occur by racialized groups.

**Study design:**

Retrospective cohort study of 3290 infants hospitalized in a single center NICU between 2017 and 2019 in the Racial and Ethnic Justice in Outcomes in Neonatal Intensive Care (REJOICE) study. Demographics and adverse social events including infant urine toxicology screening, child protective services (CPS) referrals, behavioral contracts, and security emergency response calls were collected from electronic medical records. Logistic regression models were fit to test the association of race/ethnicity and adverse social events, adjusting for length of stay. Racial/ethnic groups were compared with a White referent group.

**Results:**

There were 205 families (6.2%) that experienced an adverse social event. Black families were more likely to have experienced a CPS referral and a urine toxicology screen (OR, 3.6; 95% CI, 2.2-6.1 and OR, 2.2; 95% CI, 1.4-3.5). American Indian and Alaskan Native families were also more likely to experience CPS referrals and urine toxicology screens (OR, 15.8; 95% CI, 6.9-36.0 an OR, 7.6; 95% CI, 3.4-17.2). Black families were more likely to experience behavioral contracts and security emergency response calls. Latinx families had a similar risk of adverse events, and Asian families were less likely to experience adverse events.

**Conclusions:**

We found racial inequities in adverse social events in a single-center NICU. Investigation of generalizability is necessary to develop widespread strategies to address institutional and societal structural racism and to prevent adverse social events.

**S**tructural racism includes institutional, interpersonal, and internalized racism and contributes to racial disparities in health outcomes for minoritized groups in the US.^1-3^ Structural racism is defined as racism that is incorporated into laws, policies, practices, and procedures in institutions and systems, including healthcare, that then create unequal resources, opportunities, and exposures for racialized groups.^4^ Interpersonal racism and implicit bias can influence decisions made by health care providers and result in suboptimal health outcomes.^1^ The neonatal intensive care unit (NICU) is a space where population-level disparities are reflected, with racial and ethnic disparities in preterm birth long described.^5-7^ In fact, structural racism has been directly linked to poor perinatal and postnatal outcomes for birthing persons and their infants.^8-14^ More recent work has also highlighted racial disparities in quality of care and outcomes for families in the NICU.^15-17^

Structural racism may manifest in the NICU and contribute to racial disparities in health outcomes. Previous studies have described racial and ethnic disparities in urine toxicology screening of pregnant persons.^18-21^ Similar racial disparities have been documented in child protective services (CPS) reporting in the pediatric setting.^22^ Families and clinicians have reported unfair judgment and disparate CPS involvement by race and socioeconomic status in the NICU.^17^ Law enforcement activity in the hospital and the NICU is becoming increasingly common, has direct and indirect health effects, and places a disproportionate burden on minoritized adult and pediatric communities.^23-27^ Behavioral contracts are considered a form of policing in the hospital, because they are agreements drafted by healthcare systems used to monitor patient behaviors determined to be problematic.^28-31^ Despite the potential impact on the physical and mental health of families and patient-provider relationships, we are not aware of any studies that examine examples of policing within the NICU, including security emergency response (SER) calls or behavioral contracts.

We identified these actions by clinical teams (urine toxicology screening, CPS referrals, behavioral contracts, and SER calls) and the circumstances in which they arise as ways in which structural racism may manifest within the NICU. Because these actions may cause significant harm and are often unwanted by all parties, we defined them as adverse social events. We hypothesized that adverse social events would vary by race and ethnicity in a single tertiary care center NICU.

## Methods

This is a retrospective cohort study of 3290 infants in the Racial and Ethnic Justice in Outcomes in Neonatal Intensive Care (REJOICE) study. All infants were born in or transferred to a single tertiary care center from 2017 to 2019. All data was retrospectively collected from the electronic medical record (EMR) system. No infants were excluded for the analysis.

### Race and Ethnicity

Infant racial and ethnic data were obtained from the EMR. This information is self-reported from primary caregivers on hospital admission through the standard admission work-flow. Race and ethnicity were organized into the following groups for our analysis: Hispanic/Latinx (subsequently denoted as Latinx), non-Hispanic American Indian or Alaska Native (American Indian/Alaska Native), non-Hispanic Asian (Asian), non-Hispanic Black or African-American (Black), non-Hispanic Native Hawaiian or Pacific Islander (Native Hawaiian/Pacific Islander), non-Hispanic White (White), and other, including other race, >1 race, and not stated or unknown race.

### Covariates

Covariates were extracted from the EMR. Infant characteristics and included sex, gestational age, and birth weight. Hospitalization factors included length of stay, insurance payor, and inborn or outborn status. Birthing person characteristics included age, prenatal care, and primary language. Covariates were chosen as variables associated with NICU hospitalization, disparate care, or both.

### Adverse Social Events

After a review of the literature and discussion, author consensus was achieved to investigate the following adverse social events as a reflection of potential effects of structural racism: CPS referrals, infant urine toxicology screening, SER calls, and behavioral contracts. All adverse events were collected via chart review using search terms of notes in EMR. Key phrases such as “toxicology,” “CPS,” “security,” and “behavior” were used to identify the outcomes. Notes were reviewed by medical trainees and providers to determine if an event occurred during the initial NICU hospitalization in this single tertiary care center. Adverse social events that occurred at an outside hospital were not included as adverse events for the purpose of this study. For example, if CPS referrals were initiated at an outside hospital and communication with CPS was continued during the neonatal hospitalization at our institution, they were excluded. To address sample size limitations for the rare outcomes in the group, the rare events were combined into a combined binary variable of any adverse event.

### Statistical Analyses

Adverse social events were compared across infant and birthing person characteristics and hospital factors using a χ^2^ test for all categorical demographic predictors, a t-test for normally distributed continuous predictors and Wilcoxon Mann-Whitney test for non-normally distributed continuous predictors.

Multivariable logistic models were used to test the association of race and ethnicity with adverse social events. All models were adjusted for length of stay. *P* values of <.05 were considered statistically significant. Calculations derived from <5 infants per race and ethnicity group across multiple outcomes and rare outcomes with <50 events were excluded owing to inadequate sample size. Similarly, estimates were labeled if estimate effects were unable to be summarized secondary to inadequate sample size.

Methods and protocols for the study were approved by the Institutional Review Board of the University of California, San Francisco. STATA (version 16.1; StataCorp, College Station, TX) was used for this analysis.

## Results

### Univariate Analysis

A total of 3290 infants were admitted to the University of California, San Francisco intensive care nursery between 2017 and 2019 and were included in our study. The baseline characteristics of study participants are displayed in Table I.

Table II depicts adverse social events by infant and birthing person characteristics and hospital factors. Overall, 205 families (6.2%) experienced any adverse social event over the 3-year period. More specifically, 116 families (3.5%) experienced a CPS referral, 17 (0.52%) were presented with a behavioral contract, 40 (1.2%) were involved in an SER call, and 155 infants (4.7%) underwent a urine toxicology screen (Table II).

Table II shows adverse social events by infant and birthing person characteristics, as well as hospitalization factors. The occurrence of adverse events varied significantly by gestational age, birth weight, race and ethnicity, length of stay, insurance payor, birthing person age, and prenatal care. Infants with adverse events were more likely to have public insurance, prematurity, lower birth weights; be of Black or American Indian/Alaska Native race/ethnicity; have longer lengths of stay; and be birthing persons who were younger and spoke English. A higher proportion of infants with adverse events were outborn and birthing persons had no prenatal care (except for behavioral contracts). Adverse events also varied significantly by birth location (except for behavioral contracts and security calls) and prenatal care (with the exception of behavioral contracts). Urine toxicology screening and any adverse event varied by primary language.

Despite comprising 7.6% of the sample size, Black families were over-represented across the adverse social events: they accounted for 65% of behavioral contracts (n = 11/17), 23.3% of CPS referrals (n = 27/116), 37.5% of SERs calls (n = 15/40), 18.1% of urine toxicology screening (n = 28/ 155), and 23.4% of any adverse events (n = 48/205). Similarly, 30 individuals identified as American Indian/Alaska Native and comprised <1% of the population, yet represented 8.6% of CPS referrals (n = 10/116), 5.8% of urine toxicology screenings (n = 9/155), and 5.9% (12/205) of any adverse social event. Asian families were under-represented across all adverse events; <5% of these families had any given adverse social event, despite representing 21.2% of the study sample. Latinx and White families’ representation across all adverse events matched the study sample.

### Multivariable Analyses

To further investigate the association of racial and ethnic groups and adverse social events, multivariable analyses were conducted and adjusted for length of stay, comparing all racial and ethnic groups to the White referent group (Table III). Black families had increased odds of experiencing all adverse events compared with White families including CPS referrals, SER calls, urine toxicology screens, and any adverse social event. Behavioral contracts rarely occurred, and Black families were also more likely to experience a behavioral contract compared with White families. American Indian/Alaskan Native families were also more likely to experience CPS referrals, urine toxicology screens, and any adverse events. Latinx families did not have statistically different odds of adverse events. Asian families were statistically less likely to experience CPS referrals, urine toxicology screens, and any adverse events. For some adverse events, American Indian and Alaska Native, Native Hawaiian or Pacific Islander, and other race/ethnicity families could not be analyzed given small sample size (Table III).

## Discussion

This study investigated the variation in adverse social events, including CPS referrals, urine toxicology screening, behavioral contracts, and SER calls among racialized groups. In our study, Black and American Indian/Alaskan Native families were more likely to experience adverse events compared with White families, including SER calls and behavioral contracts, which have not been studied previously. The results of this study inform our understanding of the ways in which structural racism may contribute to racially disparate outcomes in a single-center NICU.

Our findings are consistent with previous research that has described Black pregnant persons as being at higher risk of screening for illicit drug use in the prenatal setting compared with White persons.^18-21^ This study extends our understanding to the postnatal period by evaluating urine toxicology screening of infants. Our study also highlights a higher risk of screening among American Indian and Alaskan Native families that, to our knowledge, has not been described previously. Urine toxicology screening in the perinatal and postnatal periods are important, because screening can have severe unintended consequences, such as CPS referral and infant removal from custody. In fact, Black pregnant persons are more likely to be reported to law enforcement after a positive toxicology screen.^32,33^ The American College of Obstetrics and Gynecology recommends equitable screening for substance use disorder during pregnancy and the US Preventive Services Task Force recommends universal verbal screening, without testing biological specimens for pregnant people.^34,35^ For newborn infants, there are no consensus guidelines to screen for exposure to illicit drug use; thus, screening and testing decisions are up to individual providers and institutional policies.^36^

Similar to previous studies in the pediatric setting, our study also describes disparate risk of referral to CPS for Black and American Indian and Alaska Native communities compared with White communities, and extends our understanding of disparities of referrals to the NICU.^22,33,37-40^ In a population-based study conducted in Washington from 1999 to 2013, American Indian/Alaska Native families with children <3 years of age were more often referred to CPS compared with White families, but Black families had similar rates as White families, and poverty modified the associations.^41^ Temporal, geographical, and population variations may be responsible for the differences in findings. CPS referral can result in devastating consequences, especially for Black and American Indian/Alaska Native families, who are more likely to experience separations, longer separations and foster care stays, and lower rates of unification after referrals.^22,33,37-39^ Race and ethnicity do not put children at risk of maltreatment and referral, but instead structural racism, interpersonal racism, and implicit bias increase the likelihood of reporting. As a larger system, structural racism also impacts community-level and interpersonal risk factors, and poor availability of resources for these groups.^42^

Outcomes related to policing in the hospital (SERs calls and behavioral contracts) in our cohort were rare but disproportionately affected Black families. These findings are similar to previous adult and pediatric studies describing a higher risk of SER calls and restraint use for Black families.^24-27^ Behavioral contracts are agreements drafted by healthcare providers and administrators that were initially used to promote health by advocating for medical compliance or treatment plan adherence and are increasingly used to address patient behaviors determined to be problematic for difficult families.^28-31^ In general, behavioral contracts for problematic behavior in the hospital are understudied, but further research is indicated given our findings of racial and ethnic disparities in behavioral contracts.

Our study has several limitations, including that it is a single-center study with a relatively small sample size analyzing rare events. Given that our findings are consistent with prior literature on racial disparities, the findings are unlikely to be unique to our center. However, generalization from these findings beyond our single-center data is limited. The estimates of our multivariable analysis of rare outcomes, particularly behavioral contracts, and SER calls, were unstable, indicated by wide confidence intervals, and thus compromising the estimate’s precision. Rare outcomes introduce the possibility of sparse data bias and overestimation of the effect size when using logistic regression, limitations which we acknowledge and cannot completely exclude.^43,44^ Further research, involving other institutions, will be needed to determine if our findings are replicated and extrapolated elsewhere. As a retrospective study, our study was not able to define any causal relationships. However, we can use previous literature to inform our understanding of the association between our predictor and outcomes. Finally, the outcomes collected were from search terms of notes in EMRs and thus measurement bias may occur if the documentation does not match reality.

Despite these limitations, the process of identifying and acknowledging local disparities in adverse social events is critical to achieving true equity-focused quality improvement.^45^ Recently, scholars have described ways in which providers, hospitals, and hospital systems can promote antiracism and social justice in the NICU, and have described understanding quality metrics stratified by race/ethnicity, and primary language (or REaL data) as a first step.^46^ Our study was a humble inquiry toward this first step, not designed to find statistical significance or generate blame, but to instead critically evaluate how we as providers and hospital systems may perpetuate interpersonal and structural racism and contribute to racial disparities. This study prompted deep internal reflection in our center at all levels, and in our opinion, taking part in this process by all individuals, institutions, and systems will promote health equity. We believe our methods can be used as a model for other centers who hope to interrogate their local data to support action to promote antiracism and racial equity.

Further research and advocacy involving community engagement is needed to further understand the context surrounding adverse social events in the NICU, to guide policy and practice changes, and address the upstream impact of systemic inequities to support families while reducing racial disparities.

## Figures and Tables

**Table I.** Sample characteristics

Variables	No. (%)	Mean (SD, range)
Sex		
Female	1448 (44.0)	
Male	1842 (56.0)	
Gestational age		
Preterm	1391 (42.3)	
Term	1899 (57.7)	
Birth weight (g)		2726 (893, 370-5265)
Birthing person age (years)		32.3 (5.9, 15-54)
Missing	16 (0.5)	
Prenatal care		
Yes	3263 (99.2)	
No	26 (0.79)	
Length of stay (days)		19.6 (34.9, 1-366)
Inborn/outborn		
Inborn	2333 (71.1)	
Outborn	957 (28.9)	
Race and ethnicity		
American Indian and Alaska Native	30 (0.9)	
Asian	696 (21.2)	
Black	250 (7.6)	
Latinx	810 (24.6)	
Native Hawaiian or Pacific Islander	38 (1.2)	
Other	17 (0.5)	
White	1438 (43.7)	
Missing	12 (0.4)	
Language		
Primary language other than English	292 (8.9)	
English	2986 (90.7)	
Missing	13 (0.4)	
Payor		
Public insurance	1396 (42.4)	
Private insurance	1894 (57.6)	
Total, No. (%)	3290 (100)	

Missing row not included if there were no missing data.

**Table II.** Adverse social events by infant characteristics, birthing person characteristics, and hospitalization factors

Characteristics	CPS referral		Behavioral contract		SER call		Infant urine toxicology screen		Any adverse event	
	No. (%)	*P* value	No. (%)	*P* value	No. (%)	*P* value	No. (%)	*P* value	No. (%)	*P* value
Sex		.06		.46		.16		.77		.20
Female	61 (4.2)		9 (0.6)		22 (1.5)		70 (4.8)		99 (6.8)	
Male	55 (3.0)		8 (0.4)		18 (1.0)		85 (4.6)		106 (5.8)	
Gestational age		**<.001**		**<.001**		**<.001**		**<.001**		**<.001**
Preterm	71 (5.1)		16 (1.2)		32 (2.3)		87 (6.3)		121 (8.7)	
Term	45 (2.4)		1 (0.1)		8 (0.4)		68 (3.6)		84 (4.4)	
Birth weight (g)	2365.2	**<.001**	1840.6	**<.001**	2029.5	**<.001**	2511.1	**.002**	2447.7	**<.001**
Birthing person age (y)	29.5	**<.001**	28.5	**.008**	28.5	**<.001**	30.1	**<.001**	29.9	**<.001**
Prenatal care		**<.001**		.71		**.002**		**<.001**		**<.001**
Yes	96 (2.9)		17 (0.5)		37 (1.1)		134 (4.1)		181 (5.6)	
No	19 (73.1)		0 (0)		2 (7.7)		20 (76.9)		23 (88.5)	
Length of stay (days)	34.9	**<.001**	72.4	**<.001**	42.9	**<.001**	23.2	**<.001**	29.7	**<.001**
Inborn/outborn		**<.001**		.62		.90		**<.001**		**<.001**
Inborn	62 (2.7)		13 (0.6)		28 (1.2)		79 (3.4)		111 (4.8)	
Outborn	54 (5.6)		4 (0.4)		12 (1.3)		76 (7.9)		94 (9.8)	
Race and ethnicity		**<.001**		**<.001**		**<.001**		**<.001**		**<.001**
American Indian and Alaska Native	10 (33.3)		0 (0)		1 (3.3)		9 (30.0)		12 (40.0)	
Asian	4 (0.6)		1 (0.1)		3 (0.4)		8 (1.2)		10 (1.4)	
Black	27 (10.8)		11 (4.4)		15 (6.0)		28 (11.2)		48 (19.2)	
Latinx	32 (4.0)		3 (0.4)		9 (1.1)		32 (4.0)		46 (5.7)	
Native Hawaiian or Pacific Islander	0 (0)		1 (2.6)		1 (2.6)		2 (5.3)		3 (7.9)	
White	42 (2.9)		1 (0.1)		11 (0.8)		76 (5.3)		85 (5.9)	
Language		.16		.20		.15		**.005**		**.01**
Primary language other than English	6 (2.1)		0 (0)		1 (0.3)		4 (1.4)		8 (2.7)	
English	109 (3.7)		17 (0.6)		39 (1.3)		149 (5.0)		195 (6.5)	
Payor		**<.001**		**<.001**		**<.001**		**<.001**		**<.001**
Public insurance	108 (7.7)		15 (1.1)		39 (2.8)		136 (9.7)		179 (12.8)	
Private insurance	8 (0.4)		2 (0.1)		1 (0.1)		19 (1.0)		26 (1.4)	
Total No. (%)	116 (3.5)		17 (0.5)		40 (1.2)		155 (4.7)		205 (6.2)	

Bold typeface indicates statistical significance (*P* < .05).

**Table III.** Odds of social adverse events by race and ethnicity*^,†^

Raced and ethnicities^‡^	CPS referral		Infant urine toxicology screen		Any adverse event	
	OR (95% CI)	*P* value	OR (95% CI)	*P* value	OR (95% CI)	*P* value
American Indian and Alaska Native	15.8 (6.9-36.0)	**<.001**	7.6 (3.4-17.2)	**<.001**	10.2 (4.7-21.9)	**<.001**
Asian	0.2 (0.1-0.5)	**0.002**	0.2 (0.1-0.4)	**<.001**	0.2 (0.1-0.5)	**<.001**
Black	3.6 (2.2-6.1)	**<.001**	2.2 (1.4-3.5)	**.001**	3.5 (2.4-5.2)	**<.001**
Latinx	1.3 (0.8-2.0)	.33	0.7 (0.5-1.1)	0.14	0.9 (0.6-1.3)	0.60
Native Hawaiian or Pacific Islander	§	§	1.0 (0.2-4.2)	1.0	1.3 (0.4-4.4)	.66
White	Referent group		Referent group		Referent group	

Bold typeface indicates statistical significance *P* < .05.

* Adjusted for length of stay.

† Outcomes with <50 events were excluded from analysis.

‡ "Other" race and ethnicity excluded owing to small sample size.

§ Unable to run effect estimate secondary to small sample size.

## Abbreviations

CPS: Child protective services
EMR: Electronic medical record
NICU: Neonatal intensive care unit
REJOICE: Racial and Ethnic Justice in Outcomes in Neonatal Intensive Care
SER: Security emergency response
